# NTF3 Correlates With Prognosis and Immune Infiltration in Hepatocellular Carcinoma

**DOI:** 10.3389/fmed.2021.795849

**Published:** 2021-12-06

**Authors:** Rongqiang Liu, Rongqi Li, Haoyuan Yu, Jianrong Liu, Shiyang Zheng, Yang Li, Linsen Ye

**Affiliations:** ^1^Department of Hepatic Surgery and Liver Transplantation Center, The Third Affiliated Hospital of Sun Yat-sen University, Guangzhou, China; ^2^Department of Hepatobiliary Surgery, The First Affiliated Hospital of Guangzhou Medical University, Guangzhou, China; ^3^Department of Hepatobiliary Surgery, Foshan Hospital of Traditional Chinese Medical, Foshan, China; ^4^Surgical and Transplant Intensive Care Unit of The Third Affiliated Hospital, The Third Affiliated Hospital of Sun Yat-sen University, Guangzhou, China; ^5^Department of Breast Surgery, The Third Affiliated Hospital of Guangzhou Medical University, Guangzhou, China

**Keywords:** NTF3, hepatocellular carcinoma, prognosis, immune infiltrates, TGCA

## Abstract

**Background:** The potential role of Neurotrophic factor-3(NTF3) in liver cancer is unknown. Therefore, we aimed to explore the clinical value of NTF3 in hepatocellular carcinoma (HCC).

**Methods:** We used a variety of databases to analyze the expression, relationship with prognosis and immune significance of NTF3 in liver cancer through bioinformatics.

**Results:** NTF3 was low expressed in HCC and was an independent prognostic factor in patients with HCC. CIBERSORT analysis indicated that NTF3 expression was positively correlated with CD4+ cells, mast cells, NK cells, macrophages and B cells in the tumor microenvironment. Furthermore, we found that NTF3 expression was negatively correlated with the immune checkpoints PD-L1, TIGIT and TIM-3. Functional network analysis revealed that NTF3 regulates HCC progression through a variety of cancer-related kinases, transcription factors and signaling pathways.

**Conclusions:** We demonstrate that NTF3 correlates with prognosis and immune infiltration in HCC.

## Introduction

Hepatocellular carcinoma (HCC) is one of the most common malignant tumors and the third most common cause of cancer-related deaths worldwide (1). According to global statistics, there are approximately 906,000 newly diagnosed liver cancer patients and ~830,000 liver cancer-related deaths worldwide in 2020 (1). The incidence and mortality of liver cancer in males are much higher than that in females. Globally, the incidence of liver cancer shows a clear upward trend (1, 2). Hepatitis B virus infection is a major cause of liver cancer in China (3). In developed countries, the number of patients with non-alcoholic fatty liver-related liver cancer is increasing (4). In recent years, the incidence of liver cancer has gradually decreased in areas with high incidence of liver cancer due to the spread of hepatitis B vaccine, but in some low-incidence countries, the incidence of liver cancer has increased significantly (5). Early HCC can be treated with local resection and liver transplantation, and the 5-year survival rate can reach 70% (6). In addition, studies have shown that the recurrence rate of patients with HCC after liver resection exceeds 50%, and patients with advanced HCC have even poorer responses to therapy (7, 8). Therefore, it is very necessary to identify novel biomarkers in HCC so that we can provide better clinical treatment and improve the long-term survival rate of patients.

The neurotrophin (NT) family includes neurotrophic factor-3 (NTF3). Neurotrophins are widely distributed in the brain and spinal cord, and play an important role in the formation and development of the nervous system. Neurotrophins are reported to be not only distributed in the nervous system but also significantly expressed outside the nervous system and may be associated with tumor progression in multiple cancers (9, 10). As a member of the neurotrophin family, NTF3 has a wide range of functions, including promoting the growth, development, maintaining morphology, regulating functions and repairing damaged neurons (11). It has previously been reported that NTF3 is involved in the progression of pancreatic cancer (12, 13). To date, only a few studies have reported the relationship between NTF3 expression and HCC (14, 15). However, the specific mechanism of NTF3 in HCC is still unclear.

In this study, we firstly used HCCDB database to analyze the differential expression of NTF3 mRNA in liver cancer and normal tissues and further used databases to analyze the correlation between NTF3 expression and liver cancer prognosis and tumor immune infiltration. Our results indicate that NTF3 is under-expressed in liver cancer, which is closely related to poor prognosis. Further analysis found that NTF3 was associated with tumor immunity. This study is the first bioinformatic analysis to explore the relationship between NTF3 and tumor immunity in HCC. The results help us better understand the role of NTF3 in HCC and lay a foundation for further studies.

## Materials and Methods

### HCCDB Analysis

HCCDB (http://lifeome.net/database/hccdb) is an integrative molecular database of HCC that contains 15 public HCC gene expression datasets, including data from Gene Expression Omnibus (GEO), the Liver Hepatocellular Carcinoma Project of The Cancer Genome Atlas (TCGA-LIHC) and the Liver Cancer—RIKEN, JP Project from the International Cancer Genome Consortium (ICGC LIRI-JP) (16). HCCDB can be used to perform and visualize the results of a variety of computational analyses, such as differential expression analysis, tissue-specific and tumor-specific expression analysis, survival analysis and co-expression analysis. The data for analyzing NTF3 expression between HCC tissues and adjacent normal tissues were derived from HCCDB.

### Immunohistochemistry of NTF3 Expression

The immunohistochemical analysis was performed on 80 paraffin-embedded hepatocellular carcinoma and its adjacent tissues from the Third Affiliated Hospital of Sun Yat-sen University. The tissue samples were collected from patients who underwent hepatectomy between January 2013 and January 2019. For immunohistochemistry, sections were dewaxed, hydrated and repaired with ethylenediamine tetraacetic acid (pH 8.0). The sections were then sealed with peroxidase blocker for 10 min. Sections were treated overnight at 4°C using primary antibodies (NTF3, 1:1000). The samples were washed with phosphate buffered saline and incubated with the secondary antibody at 37°C for 30 min. Two experienced pathologists independently scored the staining. The immunochemical score was based on the multiplication of the staining intensity and the number of positive staining cells. A score >3 was considered as high expression, and a score lower than 3 was considered as low expression. The ethics committee of the Third Affiliated Hospital of Sun Yat-sen University approved our study, and all patients signed an informed consent form.

### Survival Analysis

The online database Gene Expression Profiling Interactive Analysis (GEPIA) (http://gepia.cancer-pku.cn/index.html) was used to analyze the relationship between NTF3 expression and the prognosis and clinicopathological characteristics of patients with HCC (17).

### NTF3 Expression in Different Tumor Subgroups

UALCAN (http://ualcan.path.uab.edu) provides information on gene expression, survival, and epigenetic regulation using data from the TCGA database from 31 cancer types (18). The expression of NTF3 was compared between different subgroups based on patient age, patient sex, nodal metastasis status, cancer stage, TP53 mutation status and promoter methylation level.

### Co-expressed Genes and Regulatory Networks of NTF3

To investigate genes associated with NTF3, we employed the LinkedOmics database (http://www.linkedomics.org/login.php) (19). Genes co-expressed with NTF3 were analyzed statistically by calculating Pearson's correlation coefficient, and the results are presented as volcano plots and heat maps. Moreover, Gene ontology biological process (GOBP) and Kyoto Encyclopedia of Genes and Genomes (KEGG) pathway analyses and analysis of kinases, miRNAs and transcription factors targeted by NTF3 were performed with a cutoff of FDR <0.05 and 1,000 simulations.

### NTF3 Expression in Tumor-Infiltrating Immune Cells in HCC

The proportions of TIICs in HCC were estimated by estimating the relative expression of cell type-specific subsets of RNA transcripts via CIBERSORT, a deconvolution algorithm for characterizing the cell composition of complex tissues based on RNA-seq data (20). The “vioplot” package was used to visualize the proportions of 22 different TIICs between samples with high and low NTF3 expression as previously reported.

TISIDB (http://cis.hku.hk/TISIDB/index.php) is a web portal for analyzing tumor and immune system interaction that integrates multiple heterogeneous data types (21). The relationships between NTF3 expression and the gene signatures of 28 tumor-infiltrating lymphocytes (TILs) were analyzed in TISIDB.

### Statistical Analysis

Data from TCGA were processed by R-3.5.3. Overall survival (OS) and disease-free survival (DFS) were calculated by the log-rank test and Mantel-Cox test. Univariate and multivariate Cox regression models were employed to explore the prognostic role of NTF3 in HCC. Correlations between clinical characteristics and NTF3 expression were analyzed using logistic regression. Multivariate Cox analysis was used to evaluate the influence of NTF3 expression and other clinicopathological factors on survival. The correlation between NTF3 and co-expressed gene expression was measured via calculation of Pearson's correlation coefficient. *P* < 0.05 was considered statistically significant.

## Results

### Transcription Levels of NTF3 in HCC

**Figure 1:**
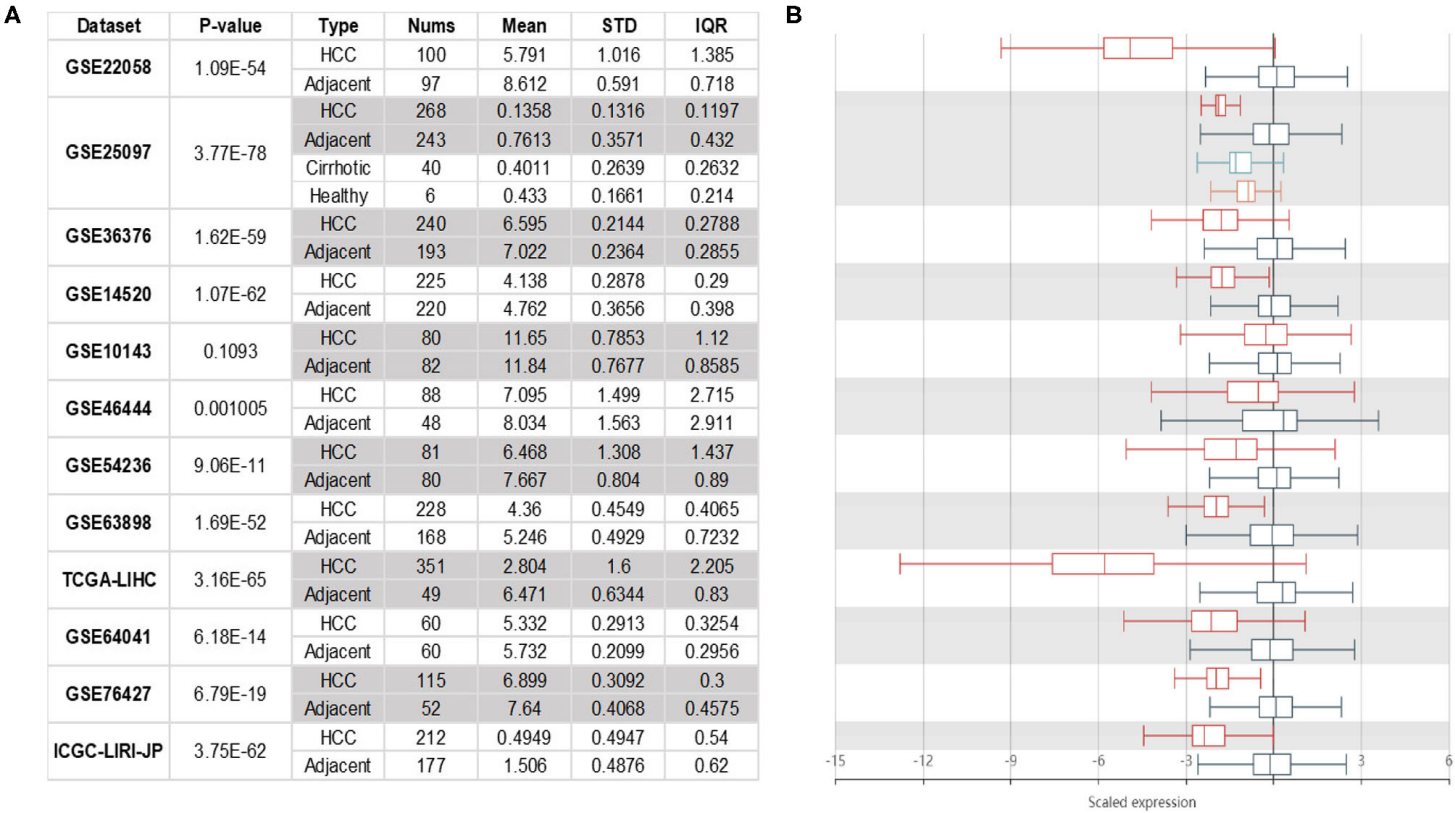
NTF3 mRNA expression in HCC. NTF3 mRNA expression in liver cancer tissues is significantly lower than normal tissues. **(A)** The table shows the specific expression of NTF3 mRNA in liver cancer tissues and normal tissues in the HCCDBs database. **(B)** The boxplot shows the comparison of the expression of NTF3 mRNA in liver cancer tissues and normal tissues in the HCCDBs database.

**Figure 2:**
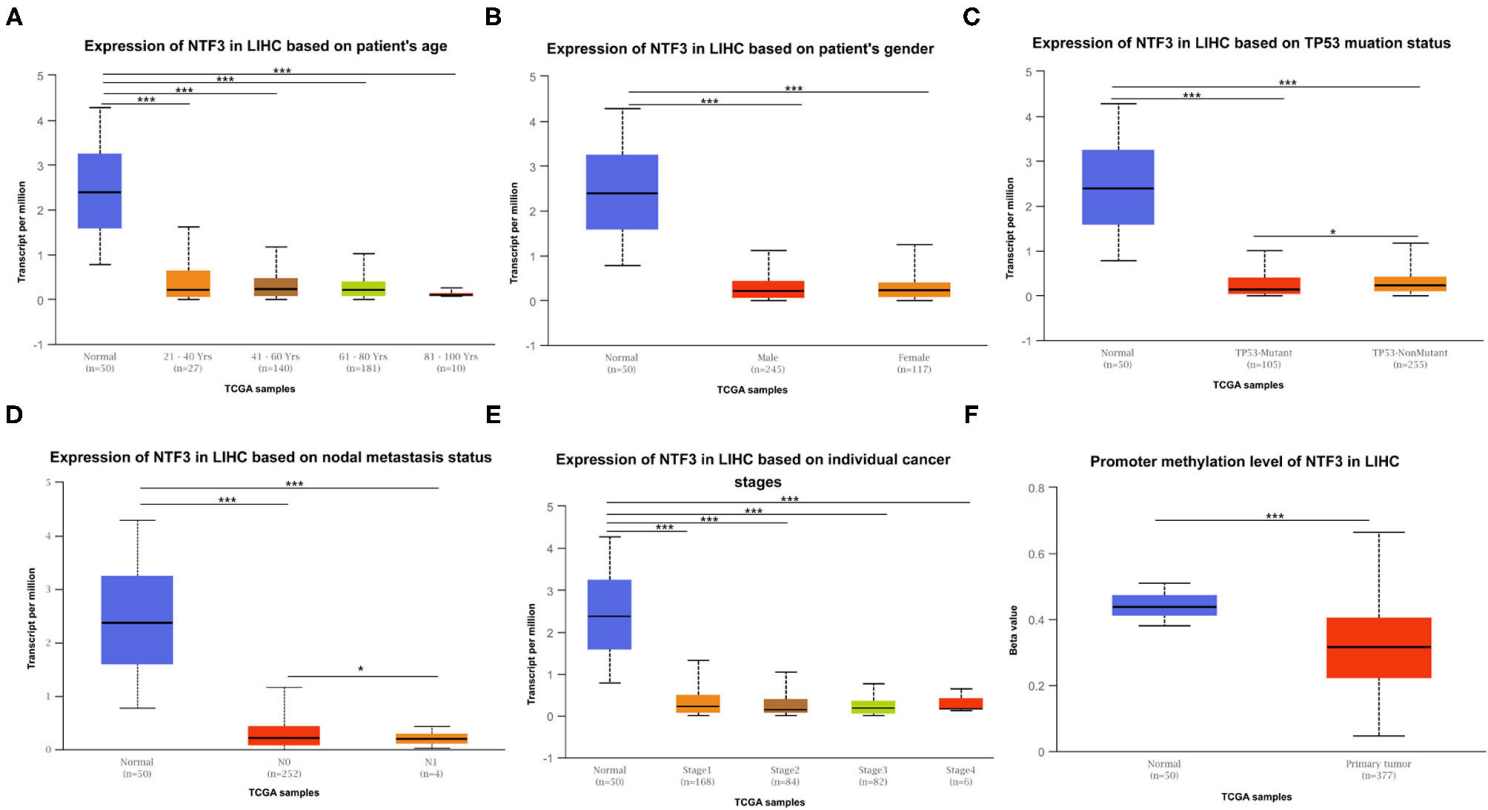
NTF3 mRNA expression varies in various subgroups of liver cancer patients based on patient age, patient sex, nodal metastasis status, cancer stage, TP53 mutation status and promoter methylation level (UALCAN). **(A)** Boxplot shows the relative expression of NTF3 in different age groups of liver cancer patients. **(B)** Boxplot shows the relative expression of NTF3 in different gender groups of liver cancer patients. **(C)** Boxplot shows the relative expression of NTF3 in the TP53 mutation status group of liver cancer patients. **(D)** Boxplot shows the relative expression of NTF3 in the nodal metastasis status group of liver cancer patients. **(E)** Boxplot shows the relative expression of NTF3 in the cancer stage group of liver cancer patients. **(F)** Boxplot shows the relative expression of NTF3 in the promoter methylation level group of liver cancer patients. Data are mean ± SE. ^*^, *P* < 0.05; ^***^, *P* < 0.001.

**Figure 3:**
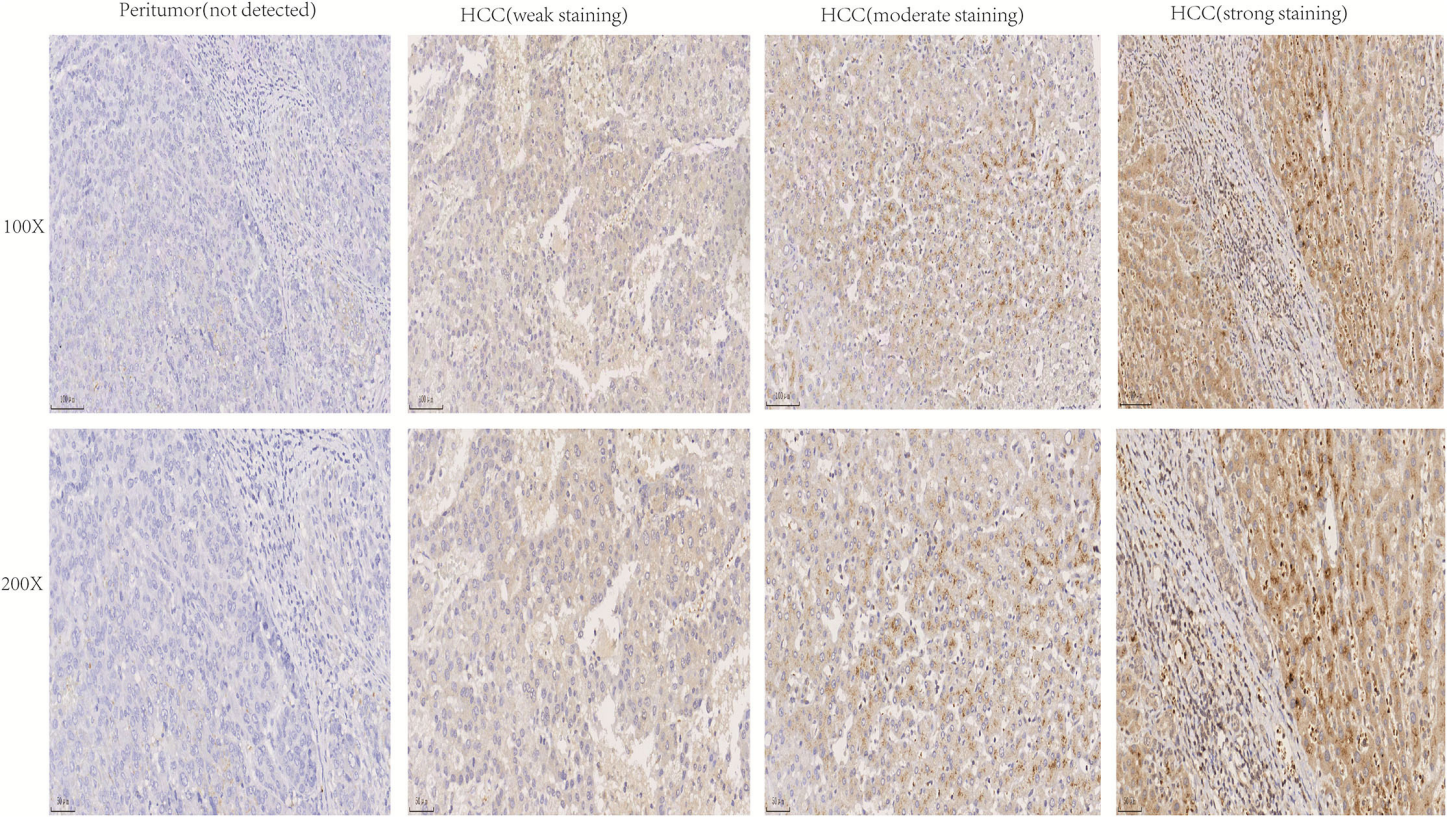
Immunohistochemical analysis of NTF3 protein expression in HCC tissues and peritumor tissues.

The expression of NTF3 mRNA between HCC tissues and normal tissues was compared in HCCDB. The results showed that NTF3 was decreased in HCC tissues compared with adjacent normal tissues (Figure 1). Furthermore, the expression of NTF3 was significantly lower in HCC subgroups stratified by age, sex, nodal metastasis status, cancer stage, tumor protein P53 (TP53) mutation status and promoter methylation level (Figure 2). Interestingly, we also found a higher level of NTF3 in TP53-wild-type HCC than in TP53-mutated HCC. In addition, the immunohistochemical results displayed positive staining of NTF3 primarily in the cytoplasm. The results showed that the majority of samples [59 cases (73.75%)] showed low expression, while a few samples [21 cases (26.25%)] showed high expression in HCC cohort. Immunohistochemical staining results were shown in Figure 3. Through the chi-square test, we found that the expression level of NTF3 in liver cancer tissue was significantly lower than the peritumor tissue (Table 1). Thus, low NTF3 expression may be a potential diagnostic indicator for HCC.

**Table 1:** The expression level of NTF3 in HCC and adjacent peritumor tissue.

**Immunohistochemical grade**	**Peritumor tissue (*N* = 80, %)**	**HCC tissue (*N* = 80, %)**	***P* value**
Negative	13 (16.25%)	31 (38.75%)	<0.01
Weak	23 (28.75%)	28 (35.0%)	
Moderate	16 (20.0%)	8 (10.0%)	
Strong	28 (35.0%)	13 (16.25%)	

### Survival Analysis of NTF3 in HCC

**Figure 4:**
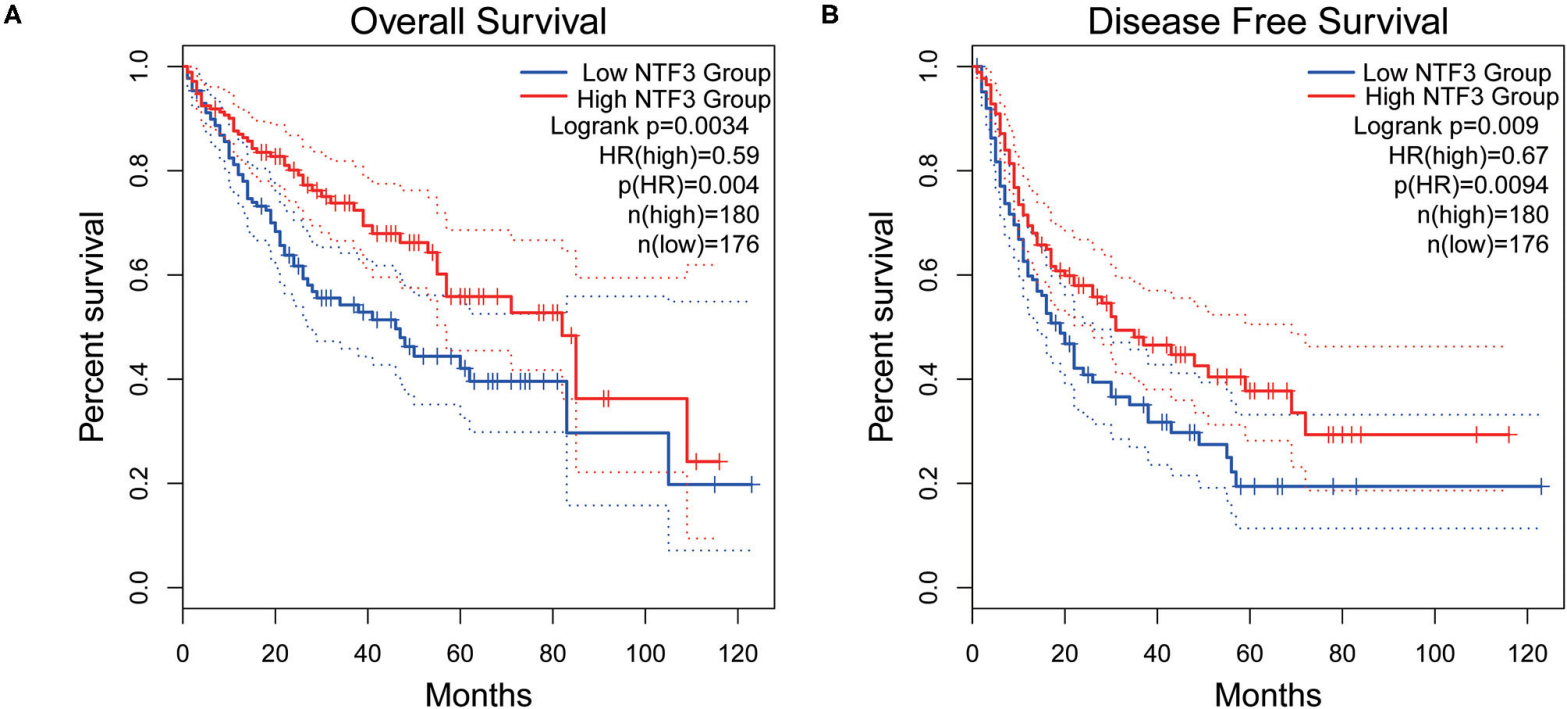
The prognostic value of NTF3 in HCC is analyzed by GEPIA. **(A)** OS, overall survival. **(B)** DFS, disease free survival.

**Figure 5:**
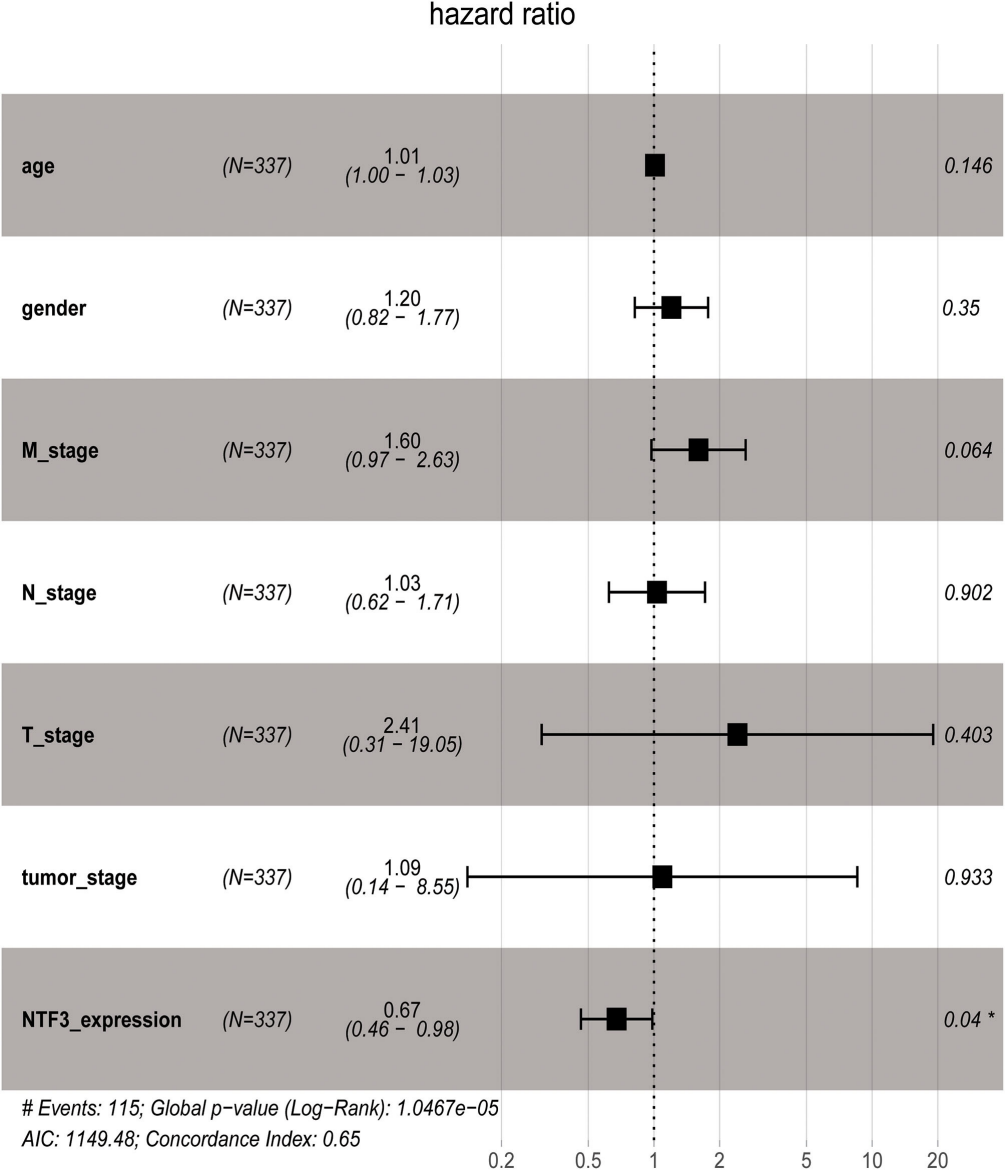
Multivariate Cox analysis of NTF3 expression and some clinical pathological factors.

We used the GEPIA database to analyze the prognostic value of NTF3 in HCC data from TCGA. HCC patients with low NTF3 expression had worse overall survival (OS) (*p* = 0.0034) and disease-free survival (DFS) (*p* = 0.009) than HCC patients with high NTF3 expression (Figure 4). Subsequent univariate Cox regression analyses revealed that sex and NTF3 expression were significantly associated with OS (Table 2). Moreover, multivariate Cox regression analysis showed that NTF3 expression was an independent prognostic factor (HR = 0.684, 95% CI 0.469–0.996, *p* = 0.048) (Figure 5). These results showed that NTF3 was an effective indicator for predicting the prognosis of HCC.

**Table 2:** Association of overall survival and clinicopathologic characteristic in TCGA.

	**Univariable Cox regression**		**Multivariate Cox regression**	
**Clinical characteristics**	**HR (95% CI)**	***P*-value**	**HR (95% CI)**	***P*-value**
Age	1.012 (0.9975–1.027)	0.106	1.0110 (0.9962–1.0261)	0.1463
Gender	1.261 (0.8647–1.838)	0.228	1.2025 (0.8167–1.7706)	0.3502
M stage	1.571 (1.044–2.365)	0.0303	1.6008 (0.9735–2.6322)	0.0637
N_stage	1.319 (0.8738–1.992)	0.187	1.0325 (0.6217–1.7145)	0.9018
T_stage	2.483 (1.707–3.614)	2.01e-06	2.4134 (0.3058–19.0493)	0.4032
Tumor_stage	2.457 (1.691–3.57)	2.42e-06	1.0929 (0.1396–8.5534)	0.9326
NTF3 expression	0.6415 (0.443–0.9291)	0.0188	0.6743 (0.4628–0.9825)	0.0402

### Networks of NTF3 Co-expressed Gene in HCC

**Figure 6:**
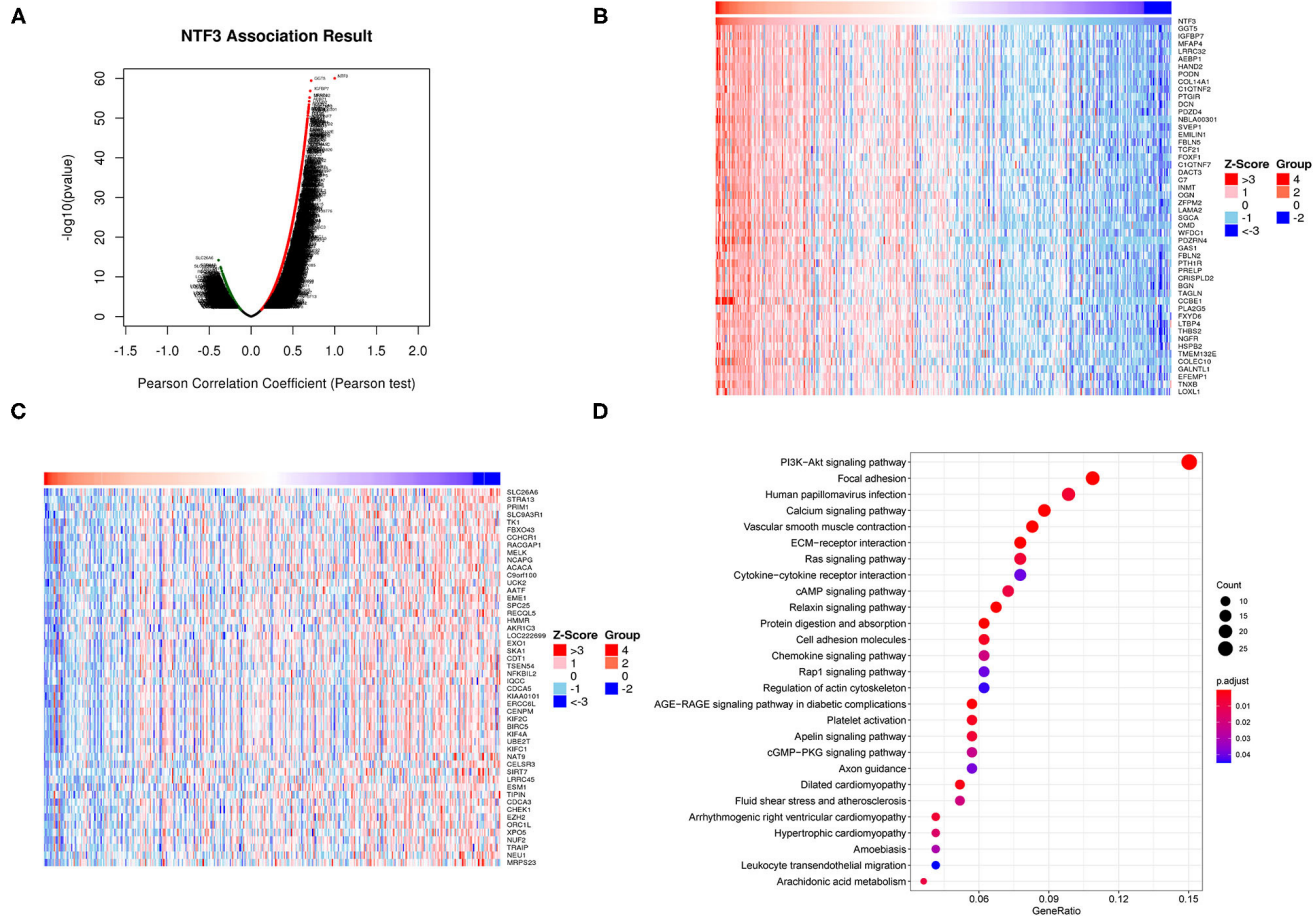
NTF3 co-expressed genes and functional enrichment (LinkedOmics). **(A)** Volcano map shows differentially expressed genes related to NTF3 in liver cancer. **(B)** The heat map shows the top 50 genes positively related to NTF3. **(B)** The heat map shows the top 50 genes positively related to NTF3. **(C)** The heat map shows the top 50 genes negatively related to NTF3. **(D)** KEGG enrichment analysis by genes co-expressed with NTF3.

To further clarify the biological significance of NTF3 in HCC, the LinkedOmics database (http://www.linkedomics.org/login.php) was employed to examine the genes co-expressed with NTF3 in the TCGA-LIHC cohort. With a cutoff of FDR <0.01.We identified 5,213 genes (red dots) that had significant positive correlations with NTF3, whereas 2,057 genes (green dots) had significant negative correlations (Figure 6A). The top 50 genes with significant positive and negative correlations with NTF3 are shown in the heat map (Figures 6B,C).

We further performed KEGG pathway analysis, and the results showed that NTF3 expression was mainly associated with the PI3K-AKT, focal adhesion, HPV infection, and calcium signaling pathways (Figure 6D). These results suggest a widespread impact of NTF3 on the transcriptome.

### NTF3-Associated Kinase, MiRNA and Transcription Factor Networks in HCC

**Figure 7:**
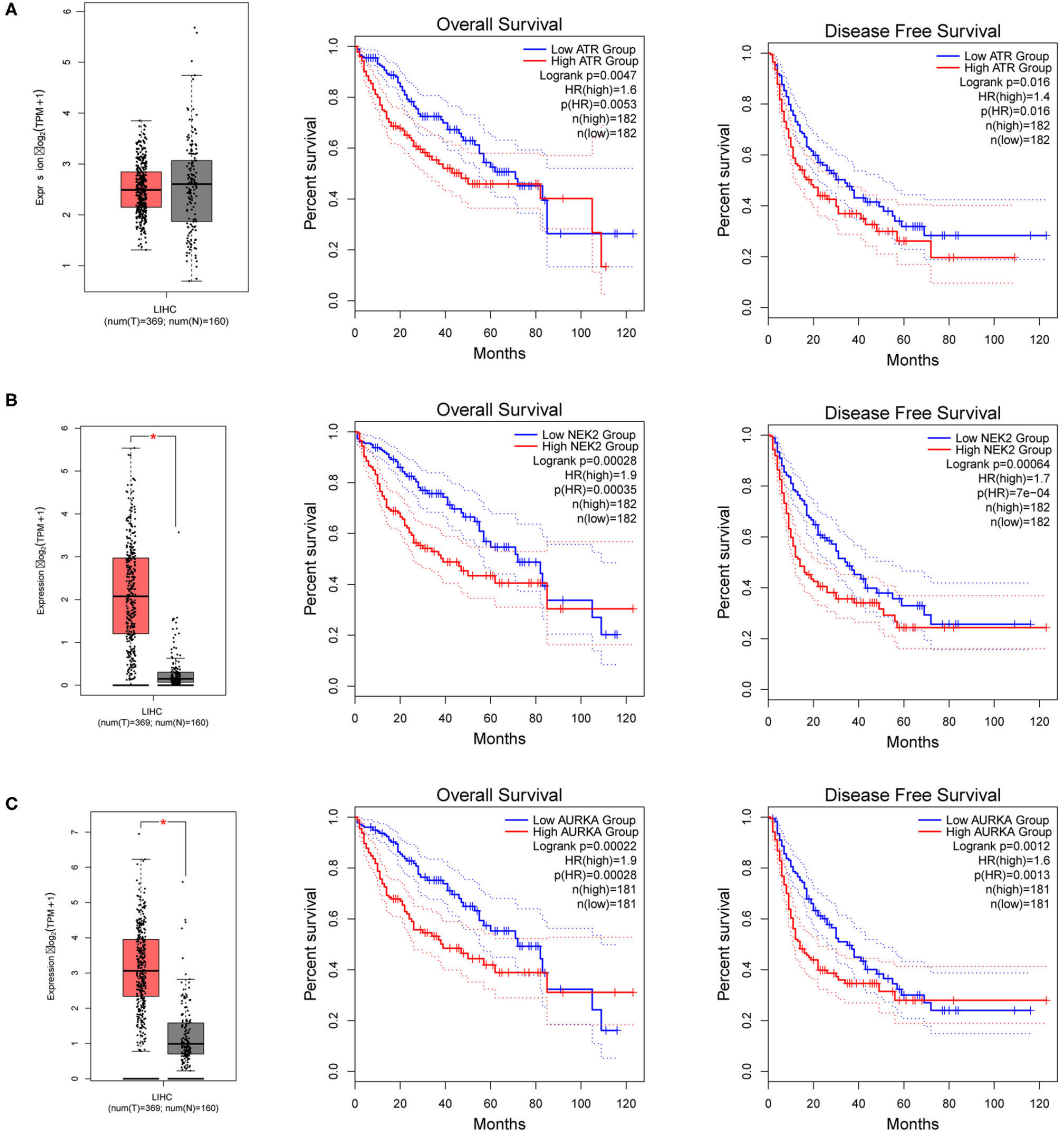
The expression and prognostic significance of NTF3-regulated kinase in HCC. **(A)** ATR, ATR serine/threonine kinase. **(B)** NEK2, NIMA-related kinase 2. **(C)** AURKA, Aurora kinase A.

To further explore the regulators of NTF3 in HCC, we analyzed the kinases, miRNAs and transcription factors associated with NTF3 co-expressed genes (Table 3). The top five most significant kinases were ATR serine/threonine kinase (ATR), checkpoint kinase 2 (CHEK2), NIMA-related kinase 2 (NEK2), ribosomal protein S6 kinase A4 (RPS6KA4), and aurora kinase A (AURKA). Among them, the expression of NEK2 and AURKA was significantly higher in HCC tissues than in adjacent normal tissues. In addition, the expression of ATR, NEK2 and AURKA was significantly associated with the OS and DFS in HCC (Figure 7).

**Table 3:** The Kinases-target networks of NTF3 in HCC.

	**Gene Set**	**Leading edge number**	**FDR**
Kinase target	Kinase_ATR	21	0
	Kinase_CHEK2	7	0.022088
	Kinase_NEK2	6	0.031257
	Kinase_RPS6KA4	11	0.03855
miRNA Target	Kinase_AURKA	13	0.039009
Transcription	TGCACGA,MIR-517A,MIR-517C	6	0.094988
Factor	CTACTGT,MIR-199A	27	0.3139
	AGCTCCT,MIR-28	21	0.31574
	AAGCAAT,MIR-137	47	0.31678
	ATACTGT,MIR-144	50	0.31716
	V$PAX5_02	6	0.050435
	CGGAARNGGCNG_UNKNOWN	9	1
	YAATNANRNNNCAG_UNKNOWN	12	0.050381
	V$PPARG_01	14	1
	V$SRF_01	19	0.009837

NTF3 was mainly associated with transcription factors related to the V$SRF_01. However, no significant miRNAs were identified by GSEA of NTF3 co-expressed genes. These results indicate a potential role of NTF3 in the progression of HCC.

### NTF3 Is Associated With the Immune Infiltration Level and T Cell Exhaustion in HCC

**Figure 8:**
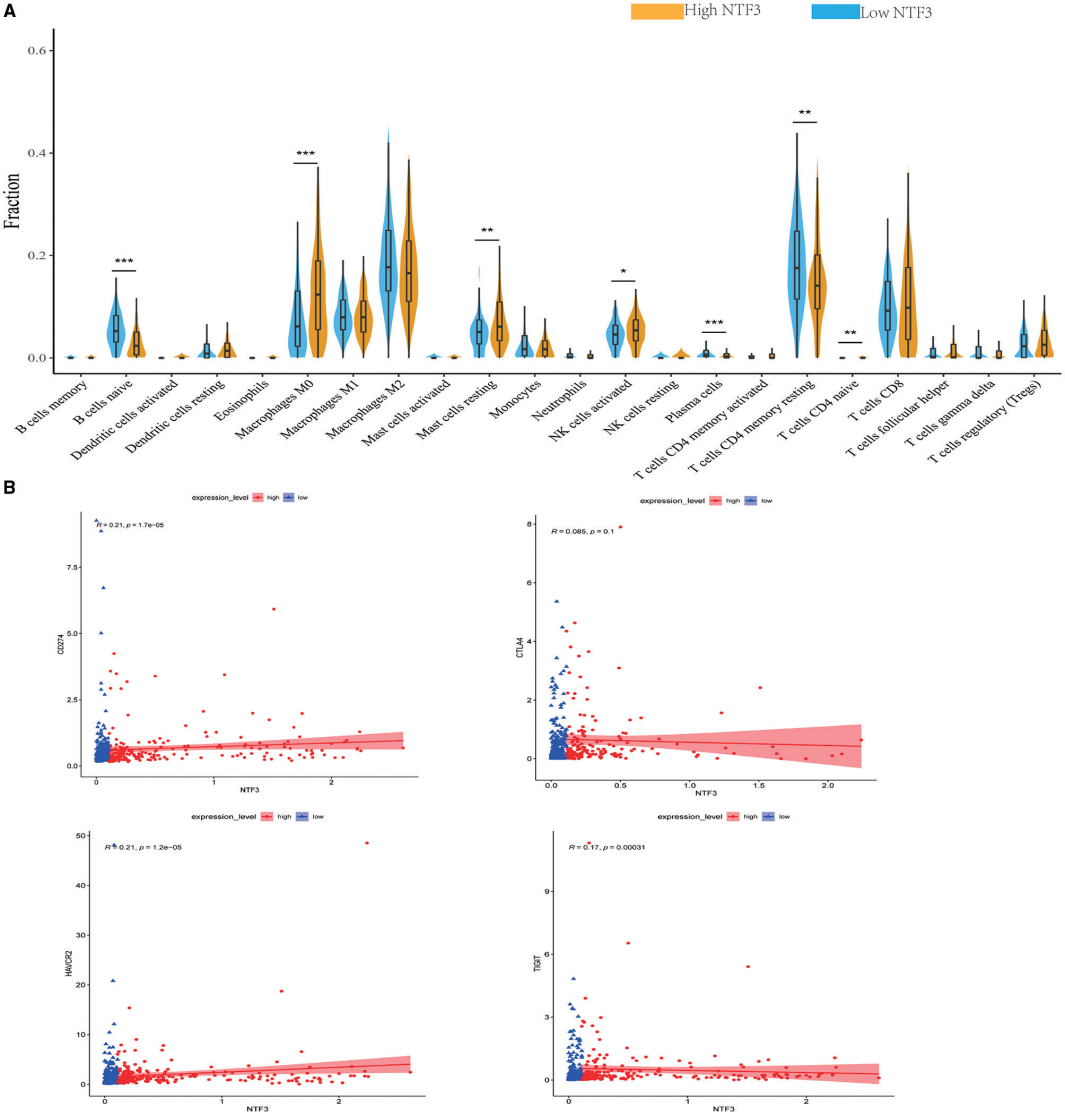
NTF3 affects immune cell infiltration and T-cell exhaustion markers (CIBERSORT) in patients with HCC. **(A)** Violin diagram shows the relationship between NTF3 expression level and the tumor-infiltrating immune cells. Among them, naïve B cells, plasma cells and resting memory CD4+ T cells are significantly increased in the low NTF3 expression group. M0 macrophages, mast cells, activated NK cells and naïve CD4+ T cells are significantly decreased in the low NTF3 group. **(B)** NTF3 expression is positively correlated with PD-L1, TIGIT and TIM-3.

We comprehensively evaluated the biological role of NTF3 in the TME through CIBERSORT (Table 4). The results showed that naïve B cells, M0 macrophages, mast cells, activated NK cells, plasma cells, resting memory CD4+T cells, and naïve CD4+T cells were the primary immune cells affected by NTF3 expression (Figure 8A). To further clarify the relationship between expression of NTF3 and different immune cells, we analyzed the association of NTF3 expression with 28 kinds of tumor-infiltrating lymphocytes (TILs) in TISIDB by conducting Spearman correlation analysis. Twenty one out of the 28 TILs were positively associated with NTF3 expression. The top five immune cell types ranked by correlation coefficient were TH1 cells (r = 0.501, *P* = 2.2e−16), mast cells (r = 0.45, *P* = 2.2e−16), NK cells (r = 0.411), macrophages (r = 0.402) and activated B cells (r = 0.402). Among these immune cells, TH1 cells showed the strongest correlation with NTF3 expression. These results demonstrated that NTF3 was obviously correlated with the immune infiltration level.

**Table 4:** Spearman correlation analysis between NTF3 and markers of immune cells in HCC.

**Terms**	**Markers**	**R**	***p*-value**
T cell exhaustion	PDCD1 (PD-1)	0.103	^*^
	CTLA4	0.085	0.110
	LAG3	0.162	^***^
	HAVCR2 (TIM3)	0.211	^***^
	GZMB	0.138	^**^
	BTLA	0.203	^***^
	CD244 (SLAMF4)	0.382	^***^
	CD274 (PD-L1)	0.207	^***^
	CD96	0.296	^***^
	IDO1	−0.065	0.180
	KDR	0.319	^***^
	PDCD1LG2 (PD-L2)	0.323	^***^
	TGFBR1	−0.048	0.323
	TIGIT	0.174	^***^
T cell (general)	CD3E	0.273	^***^
	CD3G	0.24	^***^
	CD28	0.006	0.896
	CD2	0.18	^***^
CD8 + T cells	CD8A	0.276	^***^
	CD8B	0.205	^***^
CD4 + T cells	CD4	0.459	^***^
	CD40LG (CD40L)	0.283	^***^
	CXCR4	0.274	^***^
Th1	TBX21	0.252	^***^
	STAT4	0.212	^***^
	STAT1	−0.044	0.367
	IFNG	0.096	^*^
Th2	STAT6	−0.114	^*^
	STAT5A	−0.072	0.138
Tfh	BCL6	−0.113	^*^
	IL21	0.017	0.730
Th17	STAT3	−0.213	^*^
	IL17A	0.015	0.730
Treg	FOXP3	0.021	0.665
	STAT5B	−0.189	^***^
	TGFB1	0.223	^***^
	IL2RA (CD25)	0.075	0.124
B cell	CD19	0.213	^***^
	CD79A	0.393	^***^
Monocyte	CD86(B7-2)	0.226	^***^
	CSF1R	0.295	^***^
TAM	CCL2	0.51	^***^
	CD68	0.11	^*^
	IL10	0.324	^***^
M1 Macrophage	IRF5	−0.215	^***^
	PTGS2	0.642	^***^
M2 Macrophage	CD163	0.364	^***^
	VSIG4	0.389	^***^
	MS4A4A	0.261	^***^
Neutrophils	CEACAM8	0.066	0.178
	ITGAM	0.025	0.602
	CCR7	0.299	^***^
Natural killer cell	FCGR3A(CD16)	0.24	^***^
	NCAM1(CD56)	0.464	^***^
	KIR2DL1	0.153	^**^
	KIR2DL3	0.147	^**^
	KIR2DL4	0.043	0.379
	KIR3DL1	0.151	^**^
	KIR3DL2	0.161	^***^
	KIR2DS4	0.125	^*^
Dendritic cell	HLA-DRA	0.21	^***^
	HLA-DPA1	0.272	^***^
	CD1C	0.42	^***^
	NRP1	0.048	0.326
	ITGAX	0.047	0.339

*TAM, Tumor-associated macrophages; Tfh, Follicular helper T cells; Treg, T regulatory cells*.

* *P <0.05,*

** *P < 0.01,*

*** *P < 0.001*.

Recently, immune checkpoint therapy targeting the PDL1/PD1 axis and CTLA-4 has been reported to be a promising strategy for immunotherapy of cancer. Thus, we investigated the potential influence of NTF3 expression on cancer immunotherapy and found a positive correlation between NTF3 expression and immune checkpoints, including PD-L1, TIGIT, and TIM-3, but not CTLA4 (Figure 8B). In addition, we analyzed the relationship between NTF3 and various immune infiltrating cells in the tumor microenvironment. As shown in Table 4, we found that there was significantly positively association between NTF3 expression and T cell exhaustion, T cell (general), CD8 + T cells, CD4 + T cells, Tfh, Treg, B cell, Monocyte, M2 Macrophage, neutrophils, natural killer cell and dendritic cell. These results suggested that T cell exhaustion in the TME was regulated by NTF3.

## Discussion

HCC is a serious global health problem that places a huge burden on the medical system. In the next 20–30 years, the incidence of HCC will continue to rise and reach a peak around 2030 (22). Core biopsy, as a new diagnostic method, shows great potential value in judging the character of liver tumor. Two studies confirmed the feasibility of core biopsy in the diagnosis of liver tumor through staining with tumor markers such as cytokeratin-19, glypican-3 (GPC3), hepatocyte paraffin-1, arginase-1, AFP, pCEA and CD10 (23, 24). Despite continuous improvements in diagnostic and treatment methods, the prognosis of advanced liver cancer is still poor. Finding more effective prognostic markers is particularly important. In view of our limited understanding of the role of NTF3 in liver cancer, we aimed to analyze its biological function in HCC through a comprehensive analysis of open-access databases to reveal its related regulatory pathways and specific roles in tumor immunity.

In our study, we first used HCCDB database to analyze the difference in NTF3 expression between liver cancer tissues and normal tissues. We found that NTF3 mRNA expression was significantly decreased in liver cancer tissues. Further subgroup analyses confirmed that the NTF3 mRNA expression in HCC was not affected by other clinicopathological characteristics. We also found that TP53 mutation led to decreased expression of NTF3, suggesting that NTF3 may be regulated by the TP53 gene. In addition, we further confirmed that NTF3 was relatively low expressed in liver cancer tissues through immunohistochemistry. We used GEPIA to explore the prognostic value of NTF3 in HCC. The results showed that low NTF3 expression was significantly associated with unfavorable OS and DFS. Multivariate Cox regression analysis further revealed that low NTF3 expression was an important unfavorable factor affecting the prognosis of liver cancer patients. Based on the above analysis, we have sufficient reason to believe that NTF3 is a promising and effective prognostic indicator in HCC.

Co-expressed genes act synergistically in strictly regulated biological processes, and thus they can provide alternative pathways to sidestep barriers, providing advantages in adaptive evolution (25). We used the LinkedOmics database to perform co-expression and functional enrichment analyses to explore the biological process related to NTF3 and to further study the specific mechanism of NTF3 in HCC. The enrichment analysis revealed that the PI3K-AKT, focal adhesion, HPV infection and calcium signaling pathways were differentially enriched in samples with a NTF3 low expression phenotype. It is suggested that NTF3 affects tumor progression by regulating cell metabolism, proliferation, invasion, and metastasis. Moreover, our results revealed that NTF3 has a relationship with a network of kinases, such as ATR, CDK2, NEK2, RPS6KA4 and AURKA, in HCC. These kinases play an important role in maintaining DNA stability and regulating mitosis and the cell cycle. ATR is an important kinase that activates cell responses after DNA damage, blocks cell cycle progression, repairs DNA and stabilizes replication forks, and prevents apoptosis and maintains genome stability (26, 27). CDK2 mainly binds to cyclins to form an activated protein kinase complex and phosphorylates RB to drive the cell cycle and promote cell division (28). Excessive activation of CDK2 can cause premature cell replication, promote genome instability and induce tumorigenesis (29). NEK2 plays a vital role in centrosome division, spindle formation and mitosis and is a key kinase regulating cell mitosis. Its abnormal expression affects the normal progression of mitosis, induces chromosomal instability, and promotes cell cycle disorders and tumor occurrence (30). AURKA participates in centrosome replication, separation and maturation and the formation of the poles of the spindle. Abnormal AURKA expression can lead to cell chromosome aneuploidy and thus affect genomic stability (31). We hypothesized that NTF3 affects the cell cycle, cell division, DNA replication and DNA repair through these kinases in HCC. Collectively, the enrichment analysis revealed some potential mechanisms of NTF3 in tumor progression, proved the reliability of NTF3 as a prognostic factor, and suggested that NTF3 may be a potential target for HCC therapy.

The tumor microenvironment (TME) refers to the environment in which tumor cells are located during tumor formation and metastasis and is closely related to tumor cell proliferation, apoptosis and metastasis. Tumor cells can evade the destruction of the immune system by suppressing immune cells in the tumor microenvironment in a variety of ways. Much attention has been focused on the critical role of immune cells in the tumor microenvironment in HCC. Scholars have confirmed that tumor-infiltrating immune cells regulate HCC tumor progression and are closely related to the prognosis of liver cancer patients (32, 33). T cells play a key role in tumor immunity. CD8+ T cells play a major role in antitumor immunity, and CD4+ T cells exert an antitumor effect by secreting a variety of cytokines and assisting in inducing CD8+ T cells and other immune cells. Studies have pointed out that the auxiliary effects of CD4+ T cells on CD8+ cells are indispensable (34). As a subset of CD4+ T cells, CD4+ CD25+ Foxp3+ regulatory T cells have been proved to play a key role in HCC (35). Analysis showed that myeloid-derived suppressor cells could induce the production of CD4(+)CD25(+)Foxp3(+) regulatory Tcells to exert immunosuppressive function in HCC (36). Studies have pointed that CD4+ CD25+ Foxp3+ regulatory T cells play an immunosuppressive role in antitumor immunity of HCC and significantly affect the prognosis of HCC patients (37, 38). The activation of CD8+ T cells by CD4+ T cells can induce sufficient strong antitumor effects. B cells have been shown to regulate immune responses in many ways. It has been reported that the existence of CD20+ B cells in tumors contributes to the enhancement of immunotherapy effects and prognosis in tumor patients (39). NK cells are an important component of innate immunity and can initiate multiple immune responses, including promoting CD8+ T cell antitumor immunity (40). NK cell defects or abnormalities significantly affect patient prognosis (41). Macrophages are multifaceted. On the one hand, they can induce other immune cells to recruit tumor cells. On the other hand, they can enhance the invasion and drug resistance of tumor cells, helping tumor cells escape from the immune system (42). In the current study, CIBERSORT was used to analyze immune cells in the TME, and Spearman correlation analysis was performed based on gene sets for 28 TIIC subsets using HCC data from TCGA. Our results showed that NTF3 expression was significantly positively correlated with the infiltration of TH1 cells (r = 0.501), mast cells (r = 0.45), NK cells (r = 0.411), macrophages (r = 0.402) and activated B cells (r = 0.402). Therefore, NTF3 may negatively affect HCC by changing the degree of immune cell infiltration in the TME.

Previous studies have shown that the direct combination of PD-L1 and PD-1 inhibits the function of T cells, interferes with T cell clearing of tumor cells, and leads to the tumor cell immune evasion (43). Anti-PD-1 and anti-PD-L1 antibodies have been proven to be effective in a variety of tumors and to significantly prolong patient survival (44). TIGIT is a newly discovered immune checkpoint that can be expressed by a variety of immune cells. It protects tumor cells by regulating CD4+ T, CD8+ T and NK cells to produce immunosuppressive effects. TIGIT blockers can effectively restore and enhance CD8+-mediated anti-tumor immune activity (45). In addition, the combination of anti-PD1 antibodies and anti-TIGIT antibodies can significantly enhance the killing effect on the tumor (46). TIM-3 mainly inhibits the functions of CD4+ and CD8+ T cells, and helps tumor cells escape from the immune system. Moreover, TIM-3 has also been considered to have a positive regulatory function. Our study found that the expression of NTF3 in HCC was positively correlated with the immune checkpoints PD-L1, TIGIT and TIM-3. These results suggest that NTF3 may have the ability to regulate T cell responses in HCC. Moreover, these results indicated that there was significant correlation between NTF3 expression and immune checkpoint markers and immune cells in HCC. Together, these results reveal that NTF3 participates in the tumor immune microenvironment by regulating T cells in HCC.

In conclusion, we found that NTF3 expression was downregulated in HCC and that low NTF3 expression predicted poor prognosis. NTF3 may regulate HCC through different signaling pathways. In addition, NTF3 can promote HCC progression by regulating infiltrating immune cells. In summary, NTF3 is expected to be a promising prognostic biomarker for HCC patients. Our analyses provide novel insights into the potential role of NTF3 in HCC. We strongly suggest further research on the topic of NTF3 and HCC is very necessary.

## Data Availability Statement

The original contributions presented in the study are included in the article/Supplementary Material, further inquiries can be directed to the corresponding author.

## Author Contributions

LY and YL contributed to the study inception and design. RLiu, RLi, HY, and JL equally analyzed the data and wrote the manuscript. SZ contributed to the study design and study supervision. All authors approved the final version of the manuscript.

## Funding

This work was supported by grants from National Natural Science Foundation of China (82103448), Guangdong Basic and Applied Basic Research Foundation (2019A1515110654), the Fundamental Research Funds for the Central Universities (20ykpy38), China Postdoctoral Science Foundation (2019TQ0369 and 2020M672987), and China Organ Transplantation Development Foundation (No. YZLC-2021-006). The funders had no role in study design, data collection and analysis, decision to publish, or preparation of the manuscript.

## Conflict of Interest

The authors declare that the research was conducted in the absence of any commercial or financial relationships that could be construed as a potential conflict of interest.

## Publisher's Note

All claims expressed in this article are solely those of the authors and do not necessarily represent those of their affiliated organizations, or those of the publisher, the editors and the reviewers. Any product that may be evaluated in this article, or claim that may be made by its manufacturer, is not guaranteed or endorsed by the publisher.

## Abbreviations

HCC: hepatocellular carcinoma
NTF3: neurotrophic factor-3
OS: overall survival
DFS: disease-free survival
GEPIA: Gene Expression Profling Interactive Analysis
GSEA: gene set enrichment analysis
GEO: Gene Expression Omnibus
KEGG: Kyoto Encyclopedia of Genes and Genomes
TCGA: the Cancer Genome Atlas
ATR: Ataxia telangiectasia and Rad3-related
CDK2: checkpoint kinase 2
NEK2: NIMA-related kinase 2
RPS6KA4: ribosomal protein S6 kinase A4
AURKA: aurora kinase A
TP53: tumor protein P53.

## References

[1] Sung H Ferlay J Siegel RL Laversanne M Soerjomataram I Jemal A. Global cancer statistics 2020: GLOBOCAN estimates of incidence and mortality worldwide for 36 cancers in 185 countries. CA Cancer J Clin. (2021) 71:209–9. 10.3322/caac.21660 33538338

[2] Bray F Ferlay J Soerjomataram I Siegel RL Torre LA Jemal A. Global cancer statistics 2018: GLOBOCAN estimates of incidence and mortality worldwide for 36 cancers in 185 countries. CA Cancer J Clin. (2018) 68:394–424. 10.3322/caac.21492 30207593

[3] Torre LA Bray F Siegel RL Ferlay J Lortet-Tieulent J Jemal A. Global cancer statistics, 2012. CA Cancer J Clin. (2015) 65:87–108. 10.3322/caac.21262 25651787

[4] Bucci L Garuti F Lenzi B Pecorelli A Farinati F Giannini EG.. Trevisani F; Italian Liver Cancer (ITALICA) group The evolutionary scenario of hepatocellular carcinoma in Italy: an update. Liver Int. (2017) 37:259–70. 10.1111/liv.13204 27427866

[5] Petrick JL Florio AA Znaor A Ruggieri D Laversanne M Alvarez CS. International trends in hepatocellular carcinoma incidence, 1978-2012. Int J Cancer. (2020) 147:317–30. 10.1002/ijc.32723 31597196 PMC7470451

[6] Kulik L El-Serag HB. Epidemiology and management of hepatocellular carcinoma. Gastroenterol. (2019) 156:477-491.e1. 10.1053/j.gastro.2018.08.065 30367835 PMC6340716

[7] Dhir M Melin AA Douaiher J Lin C Zhen WK Hussain SM. A review and update of treatment options and controversies in the management of hepatocellular carcinoma. Ann Surg. (2016) 263:1112–25. 10.1097/SLA.0000000000001556 26813914

[8] Javan H Dayyani F Abi-Jaoudeh N. Therapy in advanced hepatocellular carcinoma. Semin Intervent Radiol. (2020) 37:466–74. 10.1055/s-0040-1719187 33328702 PMC7732574

[9] Kuznetsov VA Tang Z Ivshina AV. Identification of common oncogenic and early developmental pathways in the ovarian carcinomas controlling by distinct prognostically significant microRNA subsets. BMC Genomics. (2017) 18:692. 10.1186/s12864-017-4027-5 28984201 PMC5629558

[10] Ying J Wang J Ji H Lin C Pan R Zhou L. Transcriptome analysis of phycocyanin inhibitory effects on SKOV-3 cell proliferation. Gene. (2016) 585:58–64. 10.1016/j.gene.2016.03.023 26995654

[11] Al-Yozbaki M Acha-Sagredo A George A Liloglou T Wilson CM. Balancing neurotrophin pathway and sortilin function: Its role in human disease. Biochim Biophys Acta Rev Cancer. (2020) 1874:188429. 10.1016/j.bbcan.2020.188429 32956766

[12] Liu D Song L Dai Z Guan H Kang H Zhang Y et al. MiR-429 suppresses neurotrophin-3 to alleviate perineural invasion of pancreatic cancer. Biochem Biophys Res Commun. (2018) 505:1077–83. 10.1016/j.bbrc.2018.09.147 30314698

[13] Ohta T Numata M Tsukioka Y Futagami F Kayahara M Kitagawa H. Neurotrophin-3 expression in human pancreatic cancers. J Pathol. (1997) 181:405–12. 10.1002/(SICI)1096-9896(199704)181:4<405::AID-PATH786>3.0.CO;2-3 9196438

[14] Yang QX Liu T Yang JL Liu F Chang L Che GL. Low expression of NTF3 is associated with unfavorable prognosis in hepatocellular carcinoma. Int J Clin Exp Pathol. (2020) 13:2280–8. 33042332 PMC7539881

[15] Liu M Liu X Liu S Xiao F Guo E Qin X. Big data-based identification of multi-gene prognostic signatures in liver cancer. Front Oncol. (2020) 10:847. 10.3389/fonc.2020.00847 32547951 PMC7270198

[16] Lian Q Wang S Zhang G Wang D Luo G Tang J. HCCDB: A Database of Hepatocellular Carcinoma Expression Atlas. Genomics Proteomics Bioinformatics. (2018) 16:269–75. 10.1016/j.gpb.2018.07.003 30266410 PMC6205074

[17] Tang Z Li C Kang B Gao G Li C Zhang Z. a web server for cancer and normal gene expression profiling and interactive analyses. Nucleic Acids Res. (2017) 45:W98–W102. 10.1093/nar/gkx247 28407145 PMC5570223

[18] Chandrashekar DS Bashel B Balasubramanya SAH Creighton CJ Ponce-Rodriguez I Chakravarthi BVSK. UALCAN: A portal for facilitating tumor subgroup gene expression and survival analyses. Neoplasia. (2017) 19:649–58. 10.1016/j.neo.2017.05.002 28732212 PMC5516091

[19] Vasaikar SV Straub P Wang J Zhang B. LinkedOmics: analyzing multi-omics data within and across 32 cancer types. Nucleic Acids Res. (2018) 46:D956–63. 10.1093/nar/gkx1090 29136207 PMC5753188

[20] Gentles AJ Newman AM Liu CL Bratman SV Feng W Kim D. The prognostic landscape of genes and infiltrating immune cells across human cancers. Nat Med. (2015) 21:938–45. 10.1038/nm.3909 26193342 PMC4852857

[21] Ru B Wong CN Tong Y Zhong JY Zhong SSW Wu WC. TISIDB: an integrated repository portal for tumor-immune system interactions. Bioinformatics. (2019) 35:4200–2. 10.1093/bioinformatics/btz210 30903160

[22] Petrick JL Kelly SP Altekruse SF McGlynn KA Rosenberg PS. Future of hepatocellular carcinoma in the United States forecast through 2030. J Clin Oncol. (2016) 34:1787–94. 10.1200/JCO.2015.64.7412 27044939 PMC4966339

[23] Wu JS Feng JL Zhu RD Liu SG Zhao DW Li N. Histopathological characteristics of needle core biopsy and surgical specimens from patients with solitary hepatocellular carcinoma or intrahepatic cholangiocarcinoma. World J Gastrointest Oncol. (2019) 11:404–15. 10.4251/wjgo.v11.i5.404 31139310 PMC6522762

[24] Chen F Bao H Deng Z Zhao Q Tian G Jiang TA. Endoscopic ultrasound-guided sampling using core biopsy needle for diagnosis of left-lobe hepatocellular carcinoma in patients with underlying cirrhosis. J Cancer Res Ther. (2020) 16:1100–5. 10.4103/jcrt.JCRT_723_19 33004754

[25] Niehrs C Pollet N. Synexpression groups in eukaryotes. Nature. (1999) 402:483–7. 10.1038/990025 10591207

[26] Chen YH Jones MJ Yin Y Crist SB Colnaghi L Sims RJ. 3rd. ATR-mediated phosphorylation of FANCI regulates dormant origin firing in response to replication stress. Mol Cell. (2015) 58:323–38. 10.1016/j.molcel.2015.02.031 25843623 PMC4408929

[27] Cimprich KA Cortez D ATR. an essential regulator of genome integrity. Nat Rev Mol Cell Biol. (2008) 9:616–27. 10.1038/nrm2450 18594563 PMC2663384

[28] Sengupta S Henry RW. Regulation of the retinoblastoma-E2F pathway by the ubiquitin-proteasome system. Biochim Biophys Acta. (2015) 1849:1289–97. 10.1016/j.bbagrm.2015.08.008 26319102

[29] Kawakami M Mustachio LM Rodriguez-Canales J Mino B Roszik J Tong P. Next-Generation CDK2/9 Inhibitors and Anaphase Catastrophe in Lung Cancer. J Natl Cancer Inst. (2017) 109:djw297. 10.1093/jnci/djw297 28376145 PMC6059250

[30] Kokuryo T Yokoyama Y Yamaguchi J Tsunoda N Ebata T Nagino M. NEK2 is an effective target for cancer therapy with potential to induce regression of multiple human malignancies. Anticancer Res. (2019) 39:2251–8. 10.21873/anticanres.13341 31092416

[31] Yan M Wang C He B Yang M Tong M Long Z. Aurora-A Kinase: A potent oncogene and target for cancer therapy. Med Res Rev. (2016) 36:1036–79. 10.1002/med.21399 27406026

[32] Ye L Zhang Q Cheng Y Chen X Wang G Shi M. Tumor-derived exosomal HMGB1 fosters hepatocellular carcinoma immune evasion by promoting TIM-1+ regulatory B cell expansion. J Immunother Cancer. (2018) 6:145. 10.1186/s40425-018-0451-6 30526680 PMC6288912

[33] Ye L Li Y Tang H Liu W Chen Y Dai T. CD8+CXCR5+T cells infiltrating hepatocellular carcinomas are activated and predictive of a better prognosis. Aging (Albany NY). (2019) 11:8879–91. 10.18632/aging.102308 31663864 PMC6834425

[34] Ghiringhelli F Ménard C Martin F Zitvogel L. The role of regulatory T cells in the control of natural killer cells: relevance during tumor progression. Immunol Rev. (2006) 214:229–38. 10.1111/j.1600-065X.2006.00445.x 17100888

[35] Granito A Muratori L Lalanne C Quarneti C Ferri S Guidi M. Hepatocellular carcinoma in viral and autoimmune liver diseases: Role of CD4+ CD25+ Foxp3+ regulatory T cells in the immune microenvironment. World J Gastroenterol. (2021) 27:2994–3009. 10.3748/wjg.v27.i22.2994 34168403 PMC8192285

[36] Hoechst B Ormandy LA Ballmaier M Lehner F Krüger C Manns MP. A new population of myeloid-derived suppressor cells in hepatocellular carcinoma patients induces CD4(+)CD25(+)Foxp3(+) T cells. Gastroenterology. (2008) 135:234–43. 10.1053/j.gastro.2008.03.020 18485901

[37] Sasaki A Tanaka F Mimori K Inoue H Kai S Shibata K. Prognostic value of tumor-infiltrating FOXP3+ regulatory T cells in patients with hepatocellular carcinoma. Eur J Surg Oncol. (2008) 34:173–9. 10.1016/j.ejso.2007.08.008 17928188

[38] Li F Guo Z Lizée G Yu H Wang H Si T. Clinical prognostic value of CD4+CD25+FOXP3+regulatory T cells in peripheral blood of Barcelona Clinic Liver Cancer (BCLC) stage B hepatocellular carcinoma patients. Clin Chem Lab Med. (2014) 52:1357–65. 10.1515/cclm-2013-0878 24646790

[39] Walsh SH Rosenquist R. Immunoglobulin gene analysis of mature B-cell malignancies: reconsideration of cellular origin and potential antigen involvement in pathogenesis. Med Oncol. (2005) 22:327–41. 10.1385/MO:22:4:327 16260850

[40] Böttcher JP Bonavita E Chakravarty P Blees H Cabeza-Cabrerizo M Sammicheli S. NK Cells stimulate recruitment of cDC1 into the tumor microenvironment promoting cancer immune control. Cell. (2018) 172:1022–37.e14. 10.1016/j.cell.2018.01.004 29429633 PMC5847168

[41] Sivori S Pende D Quatrini L Pietra G Della Chiesa M Vacca P. NK cells and ILCs in tumor immunotherapy. Mol Aspects Med. (2020) 100870. 10.1016/j.mam.2020.100870 32800530

[42] Getts DR Turley DM Smith CE Harp CT McCarthy D Feeney EM. Tolerance induced by apoptotic antigen-coupled leukocytes is induced by PD-L1+ and IL-10-producing splenic macrophages and maintained by T regulatory cells. J Immunol. (2011) 187:2405–17. 10.4049/jimmunol.1004175 21821796 PMC3159828

[43] Böger C Behrens HM Mathiak M Krüger S Kalthoff H Röcken C. PD-L1 is an independent prognostic predictor in gastric cancer of Western patients. Oncotarget. (2016) 7:24269–83. 10.18632/oncotarget.8169 27009855 PMC5029700

[44] Hays E Bonavida B. YY1 regulates cancer cell immune resistance by modulating PD-L1 expression. Drug Resist Updat. (2019) 43:10–28. 10.1016/j.drup.2019.04.001 31005030

[45] Johnston RJ Comps-Agrar L Hackney J Yu X Huseni M Yang Y. The immunoreceptor TIGIT regulates antitumor and antiviral CD8(+) T cell effector function. Cancer Cell. (2014) 26:923–97. 10.1016/j.ccell.2014.10.018 25465800

[46] Hung AL Maxwell R Theodros D Belcaid Z Mathios D Luksik AS. TIGIT and PD-1 dual checkpoint blockade enhances antitumor immunity and survival in GBM. Oncoimmunology. (2018) 7:e1466769. 10.1080/2162402X.2018.1466769 30221069 PMC6136875

